# *Cryptomphalus aspersa* Mollusc Egg Extract Promotes Regenerative Effects in Human Dermal Papilla Stem Cells

**DOI:** 10.3390/ijms18020463

**Published:** 2017-02-21

**Authors:** María Teresa Alameda, Esther Morel, Concepción Parrado, Salvador González, Ángeles Juarranz

**Affiliations:** 1 Department of Biology, Faculty of Sciences, Universidad Autónoma de Madrid, Darwin, 2, 28049 Madrid, Spain; maria.alameda@uam.es (M.T.A.); morelesther@yahoo.es (E.M.); 2 Department of Pathology, Faculty of Medicine, Universidad de Málaga, Avda. Cervantes, 2, 29071 Málaga, Spain; cparrado@uma.es; 3 Dermatology Service, Memorial Sloan-Kettering Cancer Center, 1275 York Avenue, New York, NY 10065, USA; gonzals6@mskcc.org; 4 Department of Medicine and Medical Specialties, Universidad de Alcalá, 28805 Madrid, Spain

**Keywords:** fibroblasts, proliferation, cell adhesion, cell differentiation, human dermal papilla cells, *Cryptomphalus aspersa*

## Abstract

The aim of this study was to test, by an in vitro approach, whether a natural extract derived from eggs of the mollusc *Cryptomphalus aspersa* (e-CAF) that seems to present regenerative properties, can enhance the mobilization of human hair dermal papilla cells (HHDPCs) and play a role on tissue repair and regeneration. We have tested HHDPCs proliferation by the 3-[4,5-dimethylthiazol-2-yl]-2,5-diphenyltetrazolium-bromide (MTT) assay; cell migration by using a wound healing assay, as well as the modulation of the expression of cytoskeletal (F-actin and vimentin) and cell adhesion to the extracellular matrix (ECM) (vinculin and P-FAK) proteins. We also explored whether e-CAF could lead HHDPCs to keratinocytes and/or fibroblasts by evaluating the expression of specific markers. We have compared these e-CAF effects with those induced by TGFβ_1_, implicated in regulation of cell proliferation and migration. e-CAF promotes proliferation and migration of HHDPCs cells in a time- and dose-dependent manner; it also increases the migratory behavior and the expression of adhesion molecules. These results support the fact that e-CAF could play a role on skin regeneration and be used for the prevention or repair of damaged tissue, either due to external causes or as a result of cutaneous aging.

## 1. Introduction

The regenerative properties of the skin decrease with age in a process influenced by a combination of intrinsic (genetic, hormonal, and metabolic) and extrinsic, or environmental, factors [1,2]. UV radiation is particularly relevant among the extrinsic factors because of its role as a trigger for premature aging in a process known as photoaging, induced by the formation of reactive oxygen species (ROS), which change gene expression and result in collagen degradation and elastin accumulation [2,3,4,5].

Aged skin is characterized by a flattening of the dermal-epidermal junction, a marked atrophy and a loss of elasticity of the dermal connective tissue associated with a reduction and disorganization of its major extracellular matrix (ECM) components, such as collagen and other elastic fibers, proteoglycans, and glycosaminoglycans [2,3,5]. These modifications result in a loss of structural integrity of the skin as a consequence of impaired repair processes. However, when tissue damage is generated in a situation where the repair mechanisms operate efficiently, an increase in growth factors and cytokines occurs at the wound site.

Although a plethora of factors are required for successful physiological tissue repair, transforming growth factor beta (TGFβ) expression has been demonstrated throughout wound healing and shown to regulate many processes involved in tissue repair. In this sense, TGFβ has been implicated in the production of ECM, proteases, protease inhibitors, migration, chemotaxis, and proliferation of macrophages, fibroblasts of the granulation tissue, and epithelial and capillary endothelial cells [6,7]. TGFβ_1_ is also able to induce the differentiation of dermal fibroblasts toward a myofibroblastoid phenotype by increasing the expression of α-smooth muscle actin protein (α-SMA), via phosphorylation of the Smad 2/3 complex, playing an important role in fibrosis and wound healing by synthesis, deposition, and remodeling of ECM components, as well as the capacity for synthesis of growth factors/cytokines, growth factor receptors, or integrins [8,9]. In addition, stable expression of the myofibroblast phenotype also requires adhesion-dependent signals related to the activation of focal adhesion kinase (FAK) by autophosphorylation (P-FAK) mediated by integrins which specifically interact with ECM components (collagen and fibronectin). Therefore, cells respond to the mechanical properties of the surrounding ECM, according to density and stiffness, modulating adhesion sites [10,11,12]. Vinculin is another well-characterized cytoskeleton protein that forms part of focal adhesions providing a physical connection of actin filaments to ECM through talin and integrins [13]. The role of vinculin has been identified as a mechanosensing element in cell-cell and cell-ECM junctions and, therefore, it has the ability to modify intracellular signalling pathways [13,14]. In addition, this protein regulates numerous biological processes, including growth, migration, differentiation, and cell survival via their interaction with other proteins [14,15].

As it has been indicated above, regenerative properties of the skin decrease with age and, thus, the search for substances that minimize cutaneous aging has increased in the last few years [16,17]. Previous works have proved that a secretion of the mollusc *Cryptomphalus aspersa* (SCA) has antioxidant and skin regeneration properties promoting, for instance, the proliferation, migration, and survival of keratinocytes and dermal fibroblasts [18,19,20,21,22,23,24,25].

In the skin, stem cell-based therapies have been used for regenerative medicine [26]. In this context, adult mesenchymal stem cells (MSC) have restricted capability to produce a wide range of cell types; it has been proved that they can function as a source of cells for tissue repair, wound healing, and regeneration, maintaining tissue homeostasis [26,27,28]. Human hair dermal papilla cells (HHDPCs) are a specific type of MSCs located at the base of the hair follicle surrounded by the germinal epithelium and involved in its development to produce the hair from the epidermis [29,30]. HHDPCs are not only essential for the development and formation of the hair follicle, but also constitute a reservoir of cells with potential to differentiate into a wide range of cell types, such as muscle cells, adipocytes, fibroblasts and Schwann cells, among others [30,31,32,33]. Additionally, HHDPCs act as an important repository for tissue repair after injury generation, where rapid and dynamic changes in cell microenvironment induce processes of cell differentiation and phenotypic plasticity [32,33]. Moreover, several studies with animal models point out the interest in dermal papilla cells as an important cell subpopulation of the hair follicle in regenerative processes of the skin [34,35] and, additionally, HHDPCs have been postulated as a suitable source for the construction of bioengineered skin substitutes in cases where autografting is not the appropriate option due to its immunosuppressive properties, multipotentiality, and plasticity to differentiate into several mesenchymal lineages [36]. Thus, the ability to maintain or improve the capabilities of this cell population seems to be of great interest for skin regeneration.

Therefore, the aim of this study was to test, by an in vitro approach, whether the new compound e-CAF, which is a natural extract derived from eggs of the mollusc *Cryptomphalus aspersa*, can enhance the mobilization of HHDPCs and play a role on tissue repair and regeneration. For this purposes, we first evaluated the capacity of e-CAF to improve the viability and migration of HHDPCs, as well as the modulation of the expression of relevant proteins, which play a crucial role on cytoskeleton organization (F-actin and vimentin) and cell adhesion to the ECM (vinculin and P-FAK). Finally, we also explored whether the compound could lead HHDPCs to keratinocytes and/or fibroblasts by evaluating the expression of specific markers for both skin lineages. We have also compared our results with the effects induced by TGFβ_1_, implicated in both regulation of cell proliferation and migration. These data suggest that the new compound e-CAF might modulate specific mechanisms driving skin regeneration, thus prompting the future testing of its potential therapeutic suitability in vivo for the potentiation of tissue repair and prevention of aging.

## 2. Results

### 2.1. e-CAF Treatment Affects Cell Morphology and Proliferation of HHDPCs

**Figure 1:**
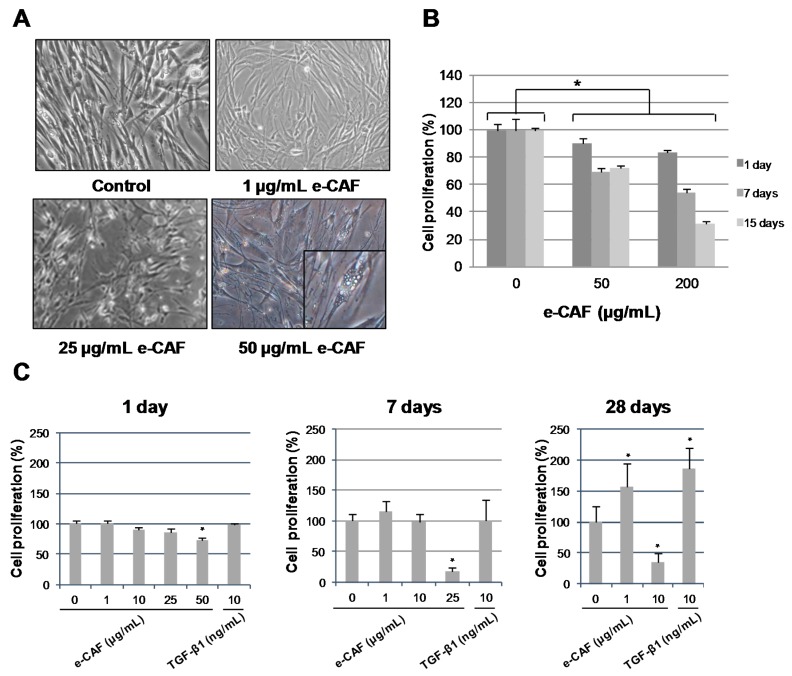
Effects of e-CAF on the morphology and proliferation of HHDPCs. (**A**) Morphological changes induced on HHDPCs by treatments with 1, 10, 25, and 50 μg/mL e-CAF for three days; phase-contrast pictures of HHDPCs from a representative experiment. Scale bar: 50 μm; The black frame shows an enlarged detail of the image. (**B**) HHDPCs proliferation after treatment with high e-CAF concentrations (50 and 200 μg/mL) for 15 days; and (**C**) HHDPCs proliferation after treatment with low concentrations of e-CAF (1, 10, and 25 μg/mL) or TGFβ_1_ (10 ng/mL) for 1, 7, and 28 days. In B and A cell proliferation, evaluated by the MTT assay, was determined as a percentage from obtained optical density values; graphs shows media and SD from quadruplicates from representative experiments; * *p* < 0.05.

We first determined whether the incubation of HHDPCs with e-CAF could affect cell morphology. As shown on Figure 1A, the morphology of cells did not change after incubation of HHDPCs with 1 or 10 μg/mL of e-CAF for one or seven days. Nonetheless, higher concentrations of the compound (25 and 50 μg/mL) resulted in an increase in cell size, long projections, and vacuolization, as well as a qualitative decrease in cell density of the treated cultures. These effects were also observed with 1 μg/mL and, particularly, with 10 μg/mL for longer incubation times (28 days). We also detected, with the incubation time and with e-CAF concentration, the presence of rounded cells and cells with long projections. In any case, however, we did not observe floating cells in the culture medium indicative of cell death.

Since we detected a qualitative decrease in cell density in the cultures, we next evaluated the effects on HHDPCs proliferation induced by different high concentrations (50 and 200 μg/mL) of e-CAF, for a variable period of time (1, 7, and 15 days); the proliferation was measured by the MTT assay (Figure 1B). As can be observed, a significant decrease in proliferation induced by e-CAF, compared to untreated cells was detected; the decrease was time- and dose-dependent, ranging from 100% to 30% in the case of cells cultured with 200 μg/mL of e-CAF for 15 days.

We also evaluated the effect of lower e-CAF concentrations (1, 10, and 25 μg/mL) (Figure 1C). Concentrations of 1 and 10 µg/mL e-CAF for one or seven days of treatment did not induce a decrease in cell proliferation, but it was significantly reduced when the treatment was of seven days with 25 μg/mL. Incubation of HHDPCs for 28 days with 1 µg/mL of e-CAF also resulted in a significant increase of cell proliferation, while a higher concentration of 10 μg/mL, for the same period of time, caused a significant decrease on cell proliferation (around 70%) (Figure 1C, right graph). TGFβ_1_ did not have any effect on cell viability when cells were treated for one or seven days in the presence of this compound with any of the evaluated concentrations (lower than 10 ng/mg), however, it significantly increased the percentage of proliferation when the incubation time was of 28 days at a concentration 10 ng/mL.

### 2.2. e-CAF Affects Cell Migration of HHDPCs

**Figure 2:**
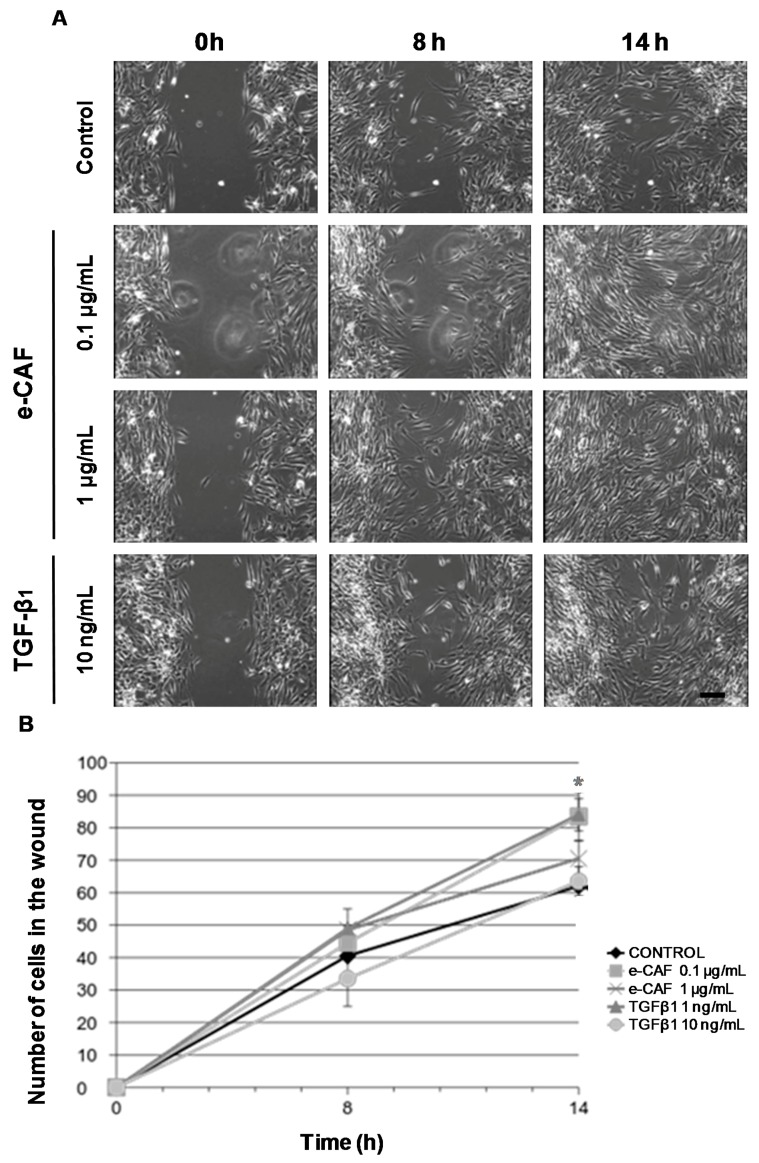
e-CAF modulates migration of HHDPCs. Wound-healing assay on HHDPCs treated with e-CAF (0.1, 0.5, 1, and 10 μg/mL) or TGFβ_1_ (1 and 10 ng/mL) for 24 h. (**A**) Panels show images taken at 0, 8, and 14 h of untreated (Control) or treated with e-CAF or TGFβ_1_ HHDPCs. Scale bar: 100 μm; and (**B**) the graph shows the media number and standard deviation (SD) of HHDPCs invading the gap area (wound) at 0, 8, and 14 h under the different experimental conditions from triplicates in a representative experiment; * *p* < 0.05.

In order to analyze the effect of e-CAF on HHDPC migration, we carried out a migration assay by using culture-inserts. HHDPCs were seeded into the two cell culture reservoirs of the inserts, which were then removed when cells reached confluence (0 h). Cells were then incubated with different concentrations of e-CAF (0.1 and 1 μg/mL) or TGFβ_1_ (1 and 10 ng/mL) for 24 h and images were taken each 15 min by a video microscopy (Figure 2 and Movies S1–S3). As shown in Figure 2A, incubation with 0.1 and 1 μg/mL of e-CAF for 8 and 14 h induced a larger number of cells to migrate into the gap area compared to untreated cultures (control) (see also Movies S1 and S2). Moreover, the number of HHDPCs was significantly larger, compared to the control, when cells were incubated with 0.1 µg/mL e-CAF for 14 h similarly to that observed with 1 ng/mL of TGFβ_1_ (Figure 2B and Movie S3). We did not observe significant differences in the case of the cultures treated with 10 ng/mL of TGFβ_1_ compared to controls.

### 2.3. e-CAF Modulates Cytoskeleton Organization and Cell Adhesion

The above results indicate that e-CAF is able to modulate both cell viability and cell migration. The direct involvement of cytoskeleton on cell proliferation and survival and migration has been widely described [11,12], so we then decided to evaluate the expression pattern of F-actin and vimentin, which configure microfilaments and intermediate filaments, respectively, after incubating HHDPCs with e-CAF (three and seven days).

**Figure 3:**
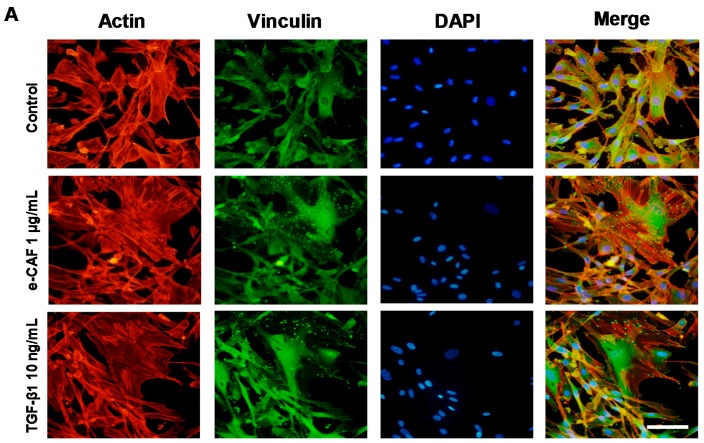

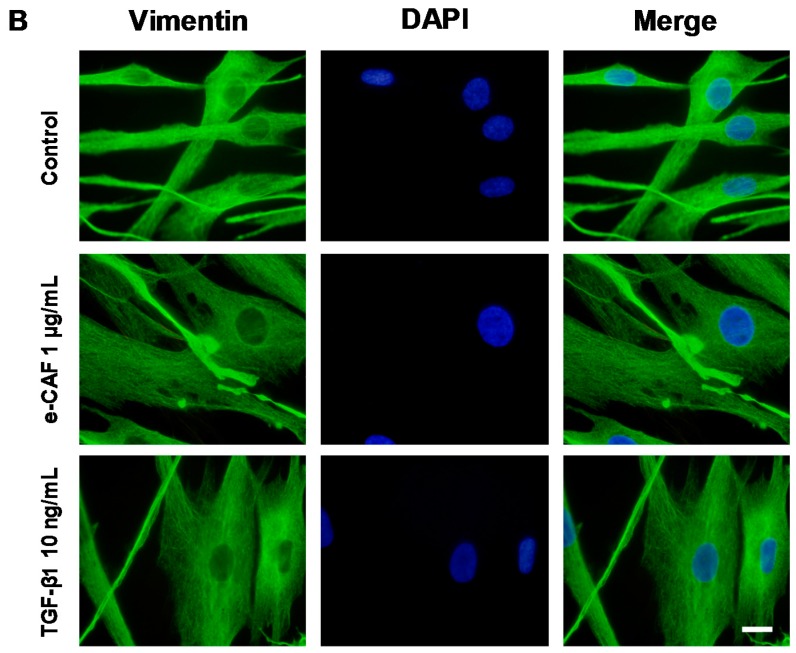
e-CAF modulates cytoskeleton organization. Immunofluorescent assays on HHDPCs treated or not (Control) with e-CAF (1 μg/mL) or TGFβ_1_ (10 ng/mL) for seven days. Vinculin (**A**) and vimentin (**B**) were determined on HHDPCs under the different experimental conditions by using specific antibodies. Actin was determined by phalloidin florescent probe (**A**). Scale bar: 50 μm (**A**) and 10 μm (**B**). DAPI, 4′-6-diamidino-2-phenylindole.

As shown in Figure 3, the incubation of HHDPCs with 1 μg/mL of e-CAF for three days induced a higher organization of stress fibers (F-actin) and vimentin intermediate filaments compared to control cells. Furthermore, these cytoskeleton modifications and morphology of treated HHDPCs seemed similar to the one found on cells incubated with 10 ng/mL TGFβ_1_ for three days (Figure 3A,B). Similar results were observed after seven days of treatment. We also decided to analyze the expression of vinculin, which is associated with actin to be part of focal adhesion complexes, in HHDPCs incubated with 1 μg/mL of e-CAF for seven days. As shown in Figure 3A, vinculin mainly accumulated in the distal regions of the cell projections on untreated cells, while incubation with e-CAF induced a different expression pattern and vinculin co-located with the ends of thick stress fibers. Similar results were observed when HHDPCs were treated with 10 ng/mL of TGFβ_1_.

Modifications of cytoskeletal proteins (actin and vimentin) and vinculin expression patterns on HHDPCs treated with e-CAF could demonstrate the influence of this compound on cell proliferation and migration processes and therefore, on cell adhesion to ECM. The involvement of these ECM proteins in cell adhesion has been described [37,38,39], so we next evaluated the expression of phosphorylated FAK protein, a tyrosine kinase which plays a critical role on cell-adhesion integrin mediated on HHDPCs treated with e-CAF seeded on different substrates of collagen I (Col I), laminin, fibronectin, and Matrigel™.

**Figure 4:**
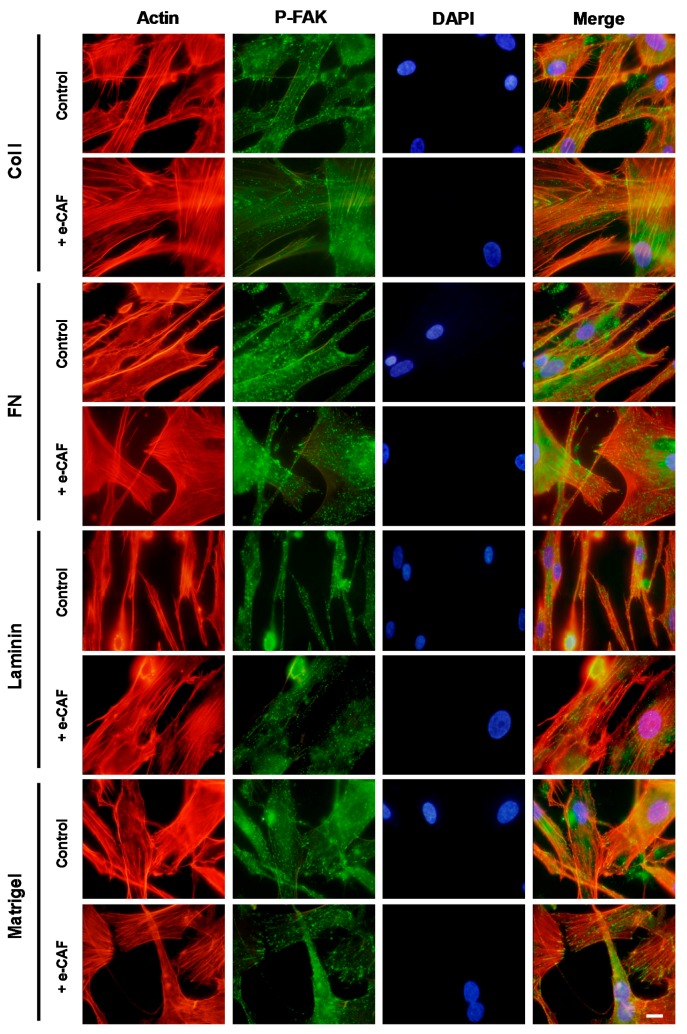
e-CAF modulates HHDPCs adhesion to ECM. Immunofluorescent assays on HHDPCs seeded on coverslips pre-treated with different substrates (collagen I, fibronectin, laminin, and Matrigel™) and treated or not (control) with e-CAF (1 μg/mL) for seven days. P-FAK and actin were determined on HHDPCs by using specific antibody and phalloidin florescent probe, respectively. Scale bar: 10 μm. DAPI, 4′-6-diamidino-2-phenylindole.

As shown in Figure 4, HHDPCs seeded on a Col I substrate enhanced the presence of stress fibers and focal contacts, which was even more obvious on cells incubated with 1 μg/mL of e-CAF for seven days. FN substrate induced the appearance of large, flattened cells with thick stress fibers co-locating with focal adhesions and, again, incubation with e-CAF increased these modifications. On the other hand, HHDPCs seeded on a laminin substrate showed a clear parallel orientation of actin which was not detected after incubation with e-CAF, and no notable changes were observed regarding focal adhesions. Finally, HHDPCs seeded on a Matrigel™ substrate induced the formation of focal contacts mostly becoming part of filopodial protrusions; structures which were even more frequent when cells were treated with e-CAF. All of these results, therefore, could suggest that ECM proteins can be crucial on HHDPCs adhesion and that e-CAF could be playing a key role on this process by enhancing it.

### 2.4. e-CAF Effects on the HHDPC Differentiation into Skin Lineages

**Figure 5:**
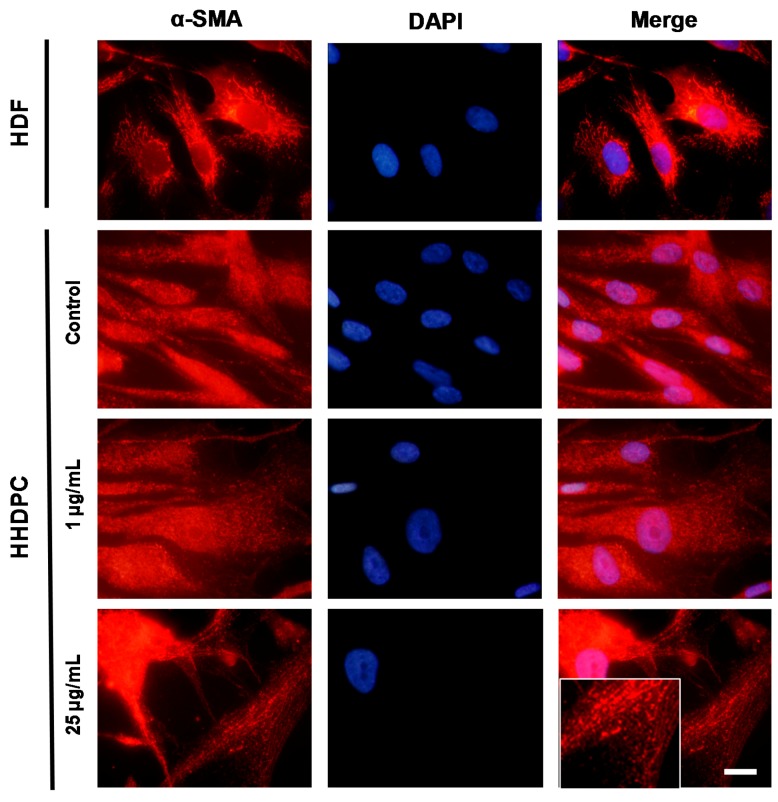
Expression pattern of α-SMA on HHDPCs treated with e-CAF. Immunofluorescent assay on HDFs and HHDPCs treated or not (control) with E-CAF (1 and 25 μg/mL) for seven days. Pictures show specific staining of α-SMA on HDFs and HHDPCs under the different experimental conditions by using specific antibodies. Scale bar: 10 μm. DAPI, 4′-6-diamidino-2-phenylindole.

**Figure 6:**
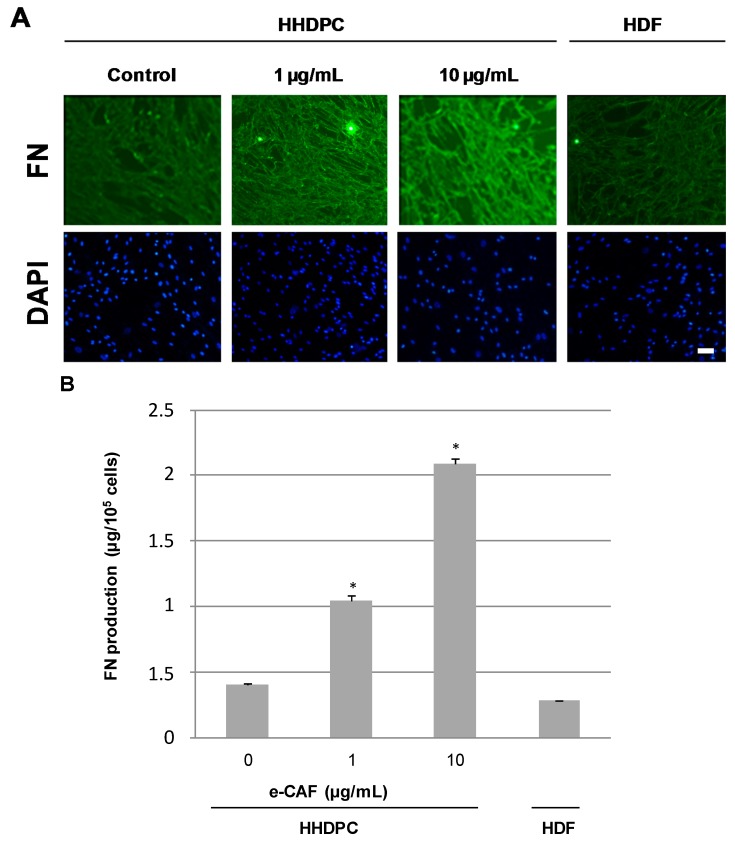
Expression and production of FN on HHDPCs treated with e-CAF. (**A**) Immunofluorescent assay on HDFs and HHDPCs treated or not (control) with e-CAF (1 and 10 µg/mL) for seven days. Pictures show specific staining of FN on HDFs and HHDPCs under the different experimental conditions by using specific antibodies. Scale bar: 50 μm. DAPI, 4′-6-diamidino-2-phenylindole; and (**B**) ELISA assay on three-day supernatants from HDFs and HHDPCs cultures. The graph shows the media and SD of FN (µg/10^5^ cells) from triplicates in HDFs and HHDPCs supernatants under the different experimental conditions in a representative experiment; * *p* < 0.05.

**Figure 7:**
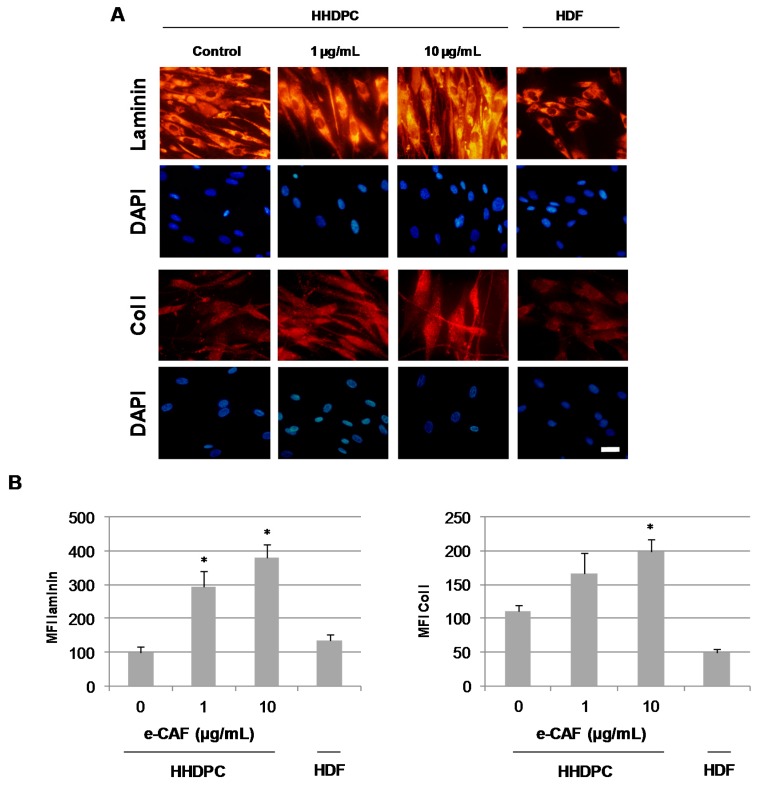
Expression and production of laminin and Col I on HHDPCs treated with e-CAF. Immunofluorescent assay on HDFs and HHDPCs treated or not (control) with e-CAF (1 and 10 µg/mL) for seven days. (**A**) Pictures show specific staining of laminin and Col I on HDFs and HHDPCs under the different experimental conditions by using specific antibodies. Scale bar: 20 μm. DAPI, 4′-6-diamidino-2-phenylindole; and (**B**) graphs show the media and SD of the median fluorescence intensity (MFI) of laminin and Col I from under the different experimental conditions in a representative experiment; * *p* < 0.05.

In order to determine whether e-CAF can promote differentiation of HHDPCs into keratinocytes and/or fibroblasts, we next explored the expression of specific markers for both skin lineages. In this sense, incubation of HHDPCs with 25 µg/mL of e-CAF for seven days induced an expression pattern of α-SMA, the specific protein for activated myofibroblasts during skin regeneration processes, similar to that found on Human dermal fibroblasts (HDFs) (Figure 5). In addition, the treatment of HHDPCs with 1 and 10 µg/mL of e-CAF for seven days induced an increased expression of fibronectin, determined by immunofluorescence and also by ELISA assay (Figure 6A,B). In addition, the incubation with 1 and 10 µg/mL of e-CAF for seven days did not change the expression patterns of laminin or Col I, but the treatment induced an increase on the mean fluorescence intensity (Figure 7A,B) of these proteins, which was statistically significant (*p* < 0.05).

**Figure 8:**
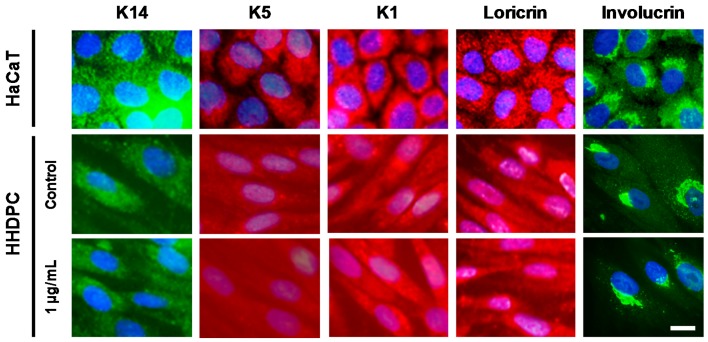
Immunofluorescence for keratinocyte specific markers: keratin 1, 5, and 14 (K1, K5, and K 14, respectively), involucrin, and loricrin on HHDPCs treated with 1 µg/mL e-CAF for seven days. The treatment did not change the expression of keratins in HHDPCs compared to keratinocyte HaCaT cells. Similar results can be observed fort loricrin and involucrin. An expression pattern of involucrin in the treated HHDPC cells was observed after e-CAF treatment, similar to that of HaCaT cells. Scale bar: 20 μm.

Finally, we evaluated the expression of keratinocyte specific markers, such as keratin 1, 5, and 14, loricrin and involucrin on HHDPCs treated with e-CAF (Figure 8). Incubation of HHDPCs with 1 µg/mL of e-CAF for seven days did not result in an expression pattern of the specific proteins for the basal (keratin 5 (K5) and keratin 14 (K14)) and spinous layer (keratin 1 (K1)) of epidermis similar to that observed on HaCaT cells. Similar results were obtained in the case of involucrin and loricrin (Figure 8, right panels). However, the HHDPC basal expression pattern of involucrin was observed to be similar to that of HaCaT cells (a diffuse staining throughout the cytoplasm and a more specific staining probably associated with the reticular system), which is also kept after the treatment with e-CAF.

## 3. Discussion

Skin aging is a natural biological process, whereby a decrease in mechanical, protective, and regenerating occurs, and clinically manifested by the appearance of xerosis, loss of elasticity, atrophy, dyschromia, and deep rhytids [2]. In addition to chronological effects, there are environmental factors which accelerate the aging process, including exposure to UV light [3,4,5]. Photoaging involves a series of events, including a reduction of the synthesis of ECM components such as collagen, increase the expression of metalloproteases that degrade the extracellular matrix, DNA damage, and decrease the number of fibroblasts [2,3,4,5].

Skin remodeling and underlying processes, such as migration, adhesion, and cell survival, are reduced as a result of skin aging. Several products from a very diverse origin, such as the secretion of mollusc *Cryptomphalus aspersa* (SCA), have been described to have regenerative properties as they can help to maintain the skin balance and delay progressive degeneration [18,19,20,23,24] and also for specific skin damage, including burns [21,22]. Actually, the effects of the new compound e-CAF from egg extracts of *C. aspersa* and another product derived from *C. aspersa* secretion, SCA, which promotes better organization of the main skin cells’ lineages, keratinocytes, and fibroblasts, are known [25]. Furthermore, it has been already described in previous investigations that positive effects of the product e-CAF on the regenerative processes are in agreement with the results obtained in this study. Thus, it is known that e-CAF also induces migration in the human keratinocyte cell line (HaCaT) and primary dermal fibroblasts and human senescent dermal fibroblasts, although no effects on cell proliferation were found; additionally, it produced an improvement in the organization of cytoskeletal proteins F-actin and vimentin and was able to promote the production of ECM components, such as FN and Col I [25]. This study shows that e-CAF could also have beneficial effects on human mesenchymal stem cells from dermal papilla (HHDPCs), improving the capabilities of this cell population, which are of great importance on skin regeneration, because of their multipotential ability to generate different skin lineages [29,30,31,32,33,34,35,36]. In fact, our results showed that treatment with 1 µg/mL of e-CAF for 28 days promoted cell proliferation and 0.1 µg/mL of e-CAF concentrations increased cell migration ability. As regards its morphology, HHDPCs treated with 1 µg/mL of e-CAF adopted a more organized arrangement parallel to its major axis associated with the overall organization of the cytoskeleton and substrate adhesion ability. A higher organization of vimentin intermediate filaments and thick stress fibers with prominent focal adhesions co-located at their ends, determined by the presence of proteins characteristic: vinculin and P-FAK are observed after e-CAF treatment.

These effects on morphology were also dependent on the type of substrate in which cells grew, since the mechanical forces exerted by the cellular microenvironment provided by the ECM are critical in regulating cell adhesion and cytoskeletal organization [37,38,39]. These effects were comparable to those induced by TGFβ_1_, and even seemed to induce some synergism when cells were grown on certain substrates, especially on FN, Col I, and Matrigel™, but not on laminin. These results are consistent with previous works, which show that FN and Col I substrates induced a greater cell adhesion in fibroblasts than on laminin; moreover, it is a remarkable fact that the FN promotes a state of high cell motility and cell growth [40]. No specific references were found regarding the behavior of these cells in 2D cultures on Matrigel™. In this regard, changes in the distribution of focal adhesions which mediate cell-ECM interactions are essential in signal transduction processes that regulate migration and cell survival [41,42].

Focal adhesions mediate ECM adhesion and are essential in signaling [15,43,44,45,46]. Thus, signal transduction through focal adhesions mediated by FAK occurs, which are involved in the regulating of key cellular processes, including mitogenic signals, survival, and cell migration [43]. Vinculin also controls these processes by modulating the interactions paxillin-FAK inducing phosphorylation of FAK, which is involved in activation of ERK [14]. As shown in this work, the dynamic regulation of focal adhesion, by evaluating vinculin and P-FAK proteins, would be of great importance not only in response to ligands immobilized as ECM proteins, but also in response to soluble ligands, such as cytokines or growth factors including TGFβ_1_ [10]. It might be possible, therefore, that e-CAF activates the same cell transduction pathways than TGFβ_1_, inducing signals which promote survival, proliferation, and cell migration. In this sense, e-CAF could be a possible source of growth factors, such as TGFβ_1_, as it has already been found in SCA, involved in tissue regeneration [25].

e-CAF seems to exert differentiation effects of HHDPCs to the main skin cell lineages toward myofibroblast, a specific fibroblastoid phenotype. Fibroblasts and other mesenchymal cells, including HHDPCs, differentiate into myofibroblasts in response to TGFβ_1_. Myofibroblasts are highly contractile cells characterized by up-regulation of proteins of the ECM, such as FN and Col I, α-SMA, and robust stress fibers and focal adhesion proteins [46]. Moreover, the dermal papilla cells have an important expression of α-SMA per se [46]; however, this expression assumed a more typical fibrillar pattern of a fibroblastoid phenotype in the presence of e-CAF. It is known that this myofibroblastoid phenotype is functionally very important for skin wound healing and requires both TGFβ_1_ and adhesion-dependent signals [8]. This synergistic effect has also been observed in the same manner in the evaluation of P-FAK on different substrates with e-CAF treatments, including FN and Col I, which induced an increase in the expression of P-FAK with respect to laminin. Therefore, specific interactions of integrin-ECM may regulate this cell adhesion-dependent differentiation pathway; additionally, there exist previous studies indicating that the basal expression of α-SMA was lower in cells cultured in laminin, compared to other ECM proteins, being higher in FN. This observation is based on the existence of an alternative splicing domain ED-A FN, which is essential for the differentiation of myofibroblasts, together with the combined action of mechanical tension and TGFβ_1_ [9,10]. All of this evidence could indicate that the molecular mechanisms implicated in the presence of TGFβ_1_ may be similar to those that occur under treatment with e-CAF, verifying that e-CAF not only promoted P-FAK expression, but was also reinforced by the substrate employed, and was even able to induce the expression of a typical α-SMA fibrillar pattern of myofibroblasts.

Additionally, the e-CAF compound appears to increase the capacity of myofibroblast replacement, from the reservoir of mesenchymal stem cells that constitute the hair follicle, acquiring a more fibroblast-like phenotype, as it is known to occur in the presence of TGF-β_1_ [47,48]; this effect is also enhanced by the employed substrate. In addition, preliminary work on its ability to induce keratinocyte migration and training of ECM was also shown. Thus, the new compound e-CAF might be capable of fine-tuning specific molecular mechanisms underlying tissue repair and regeneration. Future work will further elucidate its therapeutic and/or cosmetic potential, either preventing or treating the harmful effects resulting from aging or environmental challenges.

## 4. Materials and Methods

### 4.1. Cell Cultures

Human hair dermal papilla cells (HHDPC) (ScienCell Research Laboratories, Carlsbad, CA, USA) were cultured in mesenchymal stem cell medium (MSCM) (ScienCell Research Laboratories) supplemented with 1% (*v*/*v*) mesenchymal stem cell growth supplement (MSCGS) (ScienCell Research Laboratories), 5% (*v*/*v*) fetal bovine serum (FBS) (HyClone Laboratories, South Logan, UT, USA) and 1% (*v*/*v*) antibiotics (penicillin G (100 U/mL) and streptomycin (100 µg/mL), Fisher, Thermofisher, Madrid, Spain). For HHDPC cultures a pretreatment of the cell culture surfaces with 10 mg/mL of poly-l-lysine (ScienCell Research Laboratories) was carried out for a proper cell adhesion. Human dermal fibroblasts (HDF), derived from a skin biopsy, and the human keratinocyte cell line HaCaT were cultured in Dulbecco’s modified eagle medium (DMEM) (HyClone Laboratories) supplemented with 10% (*v*/*v*) fetal bovine serum (FBS) and 1% (*v*/*v*) antibiotics penicillin G (100 U/mL) and streptomycin (100 µg/mL) (Fisher). Cells were incubated in an incubator (HERAcell™ Heraeus, Madrid, Spain) at 37 °C, 5% humidity and 5% CO_2_.

### 4.2. Reagents

In this new revised version we have provided the method of obtaining the extract, which is also detailed below according to a US patent (US 20120107410 A1).

e-CAF (Industrial Farmaceútica Cantabria, S.A., Madrid, Spain) was prepared according to the US patent 20120107410 A1 and described by Espada et al. [25]. Briefly, e-CAF was prepared from spawn of the gastropod *Cryptomphalus aspersa*. The spawn were obtained from gastropods grown in greenhouses protected from excess light, direct rain, and aggressions by animals and insects. The gastropods were fed with fodder, water, and green plants, radishes, and vegetables, and performed their natural hibernation and reproduction cycles. During their reproductive period, snails deposit their spawn in flower-pot receptacles containing soil sieved through 2-mm-pore sieves. Once the snail spawn have been laid, the collection phase was performed at an approximate temperature of 18–24 °C and 60%–100% humidity. During this collection phase, the content of the flowerpots is deposited in 3-mm sieves, where the soil is cleaned and eliminated. The spawn were rinsed with water at a very low pressure, leaving the spawn perfectly clean. The spawn were immersed in saline solution and kept refrigerated at between 2 and 8 °C. Subsequently, the saline solution is filtered through a mesh with a diameter of less than 3 mm, such that only intact snail spawn remain. The spawn were washed with purified water and, thereafter, suspended at a concentration of 70% (*w*/*w*) in saline solution; they were lysed and homogenized by means of a Silverson grinder; filtered through a 1-mm steel mesh, and the liquid thus obtained is e-CAF, which is stored in aliquots at −20 °C, avoiding freeze-thawing cycles. The CAF is composed of organic and inorganic molecules from snail spawn, the organic molecules being polysaccharides, proteins, glycoproteins, peptides, and amino acids, where the proteins and glycoproteins present a characteristic electrophoretic pattern, and the inorganic molecules being cations and anions, such as phosphate, calcium, sodium, magnesium, iron, zinc, copper, and selenium, in their most stable ion form. e-CAF was prepared in the culture medium to the desired concentrations in a range from 0.1 to 200 μg/mL. Fresh culture medium was changed every 2–3 days to avoid product oxidation. HHDPC were treated with different concentrations of e-CAF (1, 10, 25, 50, and 200 µg/mL) or TGFβ_1_ (0.001, 0.01, 1, and 10 ng/mL) (PrepoTech, Rocky Hill, NJ, USA) during several time points (1, 7, 15, and 28 days), and then processed for the corresponding assays.

### 4.3. MTT Assay

The cell proliferation, in terms of metabolic activity, was determined by colorimetric technique MTT (3-[4,5-dimethylthiazol-2-yl]-2,5-diphenyltetrazolium-bromide) (Sigma-Aldrich, St. Louis, MO, USA). After treatments, cells were incubated with MTT (50 µg/mL) for 3 h at 37 °C and then formazan crystals were solubilized with dimethylsulfoxide (DMSO). Optical density was determined using a SpectraFluor reader plate (Tecan, Wien, Austria) at a wavelength of 542 nm.

### 4.4. Migration Assay

Cell migration was determined by wound-healing assay. HHDPCs were seeded in two cell culture reservoirs of culture-inserts (Live Cell Culture Analysis, Ibidi^®^ cells in focus, Martinsried, München, Germany) on a flat six-well plate. After 12 h, cells reached confluence and the culture-insert was removed leaving a cell-free gap (wound). Wells were then filled with culture medium supplemented with different concentrations of e-CAF (0.1, 0.5, 1 and 10 μg/mL) or TGFβ_1_ (10 ng/mL), and time-lapse microscopy was used to evaluate the wound healing process by taken images each 15 min for a total of 24 h using a digital Leica inverted microscope DMI 6000 B. Quantification of cell migration was performed by counting the cells which migrate into the gap area (wound) at different time points (0, 8, and 14 h).

### 4.5. Immunofluorescent Assays

Cells were seeded on glass coverslips (Menzel-Gläser, Braunschweig, Germany) and after 24 h were incubated with e-CAF (1, 10, and/or 25 µg/mL) or TGFβ_1_ (10 ng/mL) for seven days. After treatments, cells were fixed with 3.7% formaldehyde (Panreac, Barcelona, Spain) and then permeabilized with 0.01% Triton X-100 in PBS before the incubation with specific primary antibodies: anti-vimentin, anti-vinculin, anti-phospho-FAK, anti-α-smooth muscle actin, anti-fibronectin, anti-laminin, and anti-collagen I anti-keratin 1, 5, and 14, anti-loricrin, and anti-involucrin. Afterwards, coverslips were incubated with specific anti-IgG secondary antibody. Additionally, phalloidin was used as a specific F-actin probe (Table 1). Coverslips were mounted on slides using ProLong^®^ Gold Antifade Mountant-DAPI (Thermo Fisher Scientific, Waltham, MA, USA).

In order to analyze the effect of simultaneous treatments e-CAF and ECM proteins on the expression of adhesion and cytoskeletal proteins, HHDPC were grown on coverslips pretreated with different substrates: collagen I (Col I), fibronectin (FN), laminin, and Matrigel™, (Corning Inc., Corning, NY, USA) according of the manufacturer´s specifications.

### 4.6. ELISA Assay

HHDPC were incubated with e-CAF (1 and 10 µg/mL) for three days on six-well plates. After this time, supernatants were collected and frozen at −20 °C. The concentration of fibronectin on three-day supernatants was determined by ELISA (enzyme-linked immunosorbent assay) (Abcam, Cambridge, UK) and was compared with fibronectin concentration found on HF supernatants according to manufacturer’s recommendations using a SpectraFluor (Tecan) reader plate at a wavelength of 490 nm.

### 4.7. Data Processing and Image Analysis

Data are represented as the mean ± standard deviation (SD) of at least three independent experiments. Statistical significance was determined by statistical test of analysis of variance (ANOVA), using Statgraphics Plus 5.1. (Warrenton, VA, USA) Differences were considered to be significant at *p* < 0.05 (*). Microscopic images were obtained by using an epifluorescence microscope coupled to a CCD camera DP70 (Olympus BX-61) with UV filters for the excitation light (360–370 nm excitation filter UG-1), blue (450–490 nm excitation filter BP 490), or green (570–590 nm excitation filter 590 DM). Processing of the pictures was performed by Photoshop Extended CS5 12.0 (Adobe Systems Inc., Mountain View, CA, USA). Quantitative image analysis was performed using public domain ImageJ 1.8 software (available on: http://rsb.info.nih.gov/ij/) (Wayne Rasband National Institutes of Health, Bethesda, MD, USA).

## 5. Conclusions

e-CAF promotes proliferation and migration of HHDPCs cells in a time- and dose-dependent manner, moreover it increases the migratory behavior and the expression of adhesion molecules. e-CAF might be capable of fine-tuning specific molecular mechanisms underlying tissue repair and regeneration.

## Figures and Tables

**Table 1:** The following table shows the description of the antibodies employed and phalloidin used as a specific F-actin probe (source, manufacturer, and dilution specified by the manufacturer).

Antibody	Source	Manufacturer	Dilution
Anti-α-smooth muscle actin (α-SMA)	Rabbit (polyclonal)	Abcam^®^	1:200
Anti-Keratin 1	Rabbit (polyclonal)	Abcam^®^	1:100
Anti-Keratin 5	Rabbit (polyclonal)	Abcam^®^	1:500
Anti-Keratin 10	Mouse (monoclonal)	DAKO^®^	1:50
Anti-Keratin 14	Mouse (monoclonal)	Abcam^®^	1:200
Anti-Collagen I	Rabbit (polyclonal)	Santa Cruz^®^	1:100
Anti-Mouse IgG Alexa Fluor 488	Goat (polyclonal)	Interchim^®^	1:250
Anti-Rabbit IgG Alexa Fluor 546	Goat (monoclonal)	Interchim^®^	1:250
Anti-Mouse IgG Alexa Fluor 488	Goat (polyclonal)	Interchim^®^	1:250
Anti-Rabbit IgG Alexa Fluor 546	Goat (monoclonal)	Interchim^®^	1:250
Anti-Involucrin	Mouse (monoclonal)	Sigma-Aldrich^®^	1:50
Phalloidin Alexa Fluor 546	-	Invitrogen^®^	1:100
Anti-phospho-FAK (pY397)	Mouse (monoclonal)	BD Transduction Laboratories^®^	1:100
Anti-Fibronectin	Rabbit (polyclonal)	Abcam^®^	1:100
Anti-Laminin	Rabbit (polyclonal)	Abcam^®^	1:100
Anti-Loricrin	Rabbit (polyclonal)	Sigma-Aldrich^®^	1:100
Anti-Vimentin	Mouse (monoclonal)	Sigma-Aldrich^®^	-
Anti-Vinculin	Mouse (monoclonal)	Sigma-Aldrich^®^	1:50

## Supplementary Materials

Supplementary materials can be found at www.mdpi.com/1422-0067/18/2/463/s1.
